# Bioactive and Physico-Chemical Assessment of Innovative Poly(lactic acid)-Based Biocomposites Containing Sage, Coconut Oil, and Modified Nanoclay

**DOI:** 10.3390/ijms24043646

**Published:** 2023-02-11

**Authors:** Raluca Nicoleta Darie-Niță, Anamaria Irimia, Florica Doroftei, Laura Mihaela Stefan, Andrzej Iwanczuk, Agnieszka Trusz

**Affiliations:** 1 “Petru Poni” Institute of Macromolecular Chemistry, 41A Gr. Ghica Voda Alley, 700487 Iasi, Romania; 2 National Institute of Research and Development for Biological Sciences, 296 Splaiul Independentei, 060031 Bucharest, Romania; 3 Faculty of Environmental Engineering, Wroclaw University of Science and Technology, Wybrzeze Wyspiańskiego 27, 50-370 Wroclaw, Poland

**Keywords:** poly(lactic acid), sage, coconut oil, nanoclay, antioxidant, antimicrobial, cytocompatibility

## Abstract

The bioactivity of the versatile biodegradable biopolymer poly(lactic acid) (PLA) can be obtained by combining it with natural or synthetic compounds. This paper deals with the preparation of bioactive formulations involving the melt processing of PLA loaded with a medicinal plant (sage) and an edible oil (coconut oil), together with an organomodifed montmorillonite nanoclay, and an assessment of the resulting structural, surface, morphological, mechanical, and biological properties of the biocomposites. By modulating the components, the prepared biocomposites show flexibility, both antioxidant and antimicrobial activity, as well as a high degree of cytocompatibility, being capable to induce the cell adherence and proliferation on their surface. Overall, the obtained results suggest that the developed PLA-based biocomposites could potentially be used as bioactive materials in medical applications.

## 1. Introduction

A large variety of polyesters are used worldwide in different applications due to their valuable properties. Natural or synthetic materials can be combined with neat polyesters to improve their bioactive properties and obtain the appropriate features for various medical applications [1]. Some efficient methods considered for controlling antibiotic-resistant pathogens include the use of essential oils (EOs), bacteriocins, antibodies, nanotherapy, or quorum-sensing inhibitors [2]. Among the antibiotic resistant bacteria, *Escherichia coli* and *Staphylococcus aureus* are two species of strains responsible for clinical infections. Therefore, innovative antibacterial compounds and materials are in major demand in the public health area.

Poly(lactic acid) (PLA) is a versatile biodegradable biopolymer. It is easily synthesized from abundant renewable resources, its degradation products being non-toxic to humans and the environment. PLA is environmentally friendly, requiring 25–55% less energy than petrol-based polymers to produce [3]. Due to its advantageous characteristics, such as the ease with which it is produced and processed, its good mechanical properties, compostability, recyclability, and biocompatibility, and the fact that it has low to no disclosed carcinogenic effects, PLA is successfully used as a biomaterial in a multitude of healthcare applications, e.g., as a drug carrier, in tissue engineering, in dental care, as a cardiovascular implant, in orthopedic interventions, wound dressing, cancer therapy, or medical tools [4]. PLA is also a three-dimensionally (3D) printable biopolymer and is employed for the rapid prototyping and efficient manufacturing of 3D printed products to generate patient-specific scaffolds or medical equipment, such as the nasopharyngeal swabs or personal protective equipment (PPE) often used during the coronavirus (COVID-19) pandemic [1]. Nevertheless, some polyesters, such as PLA, polyhydroxybutyrate (PHB), and poly(ε-caprolactone) (PCL), cannot be used in neat form for biomedical purposes due to their lack of bioactivity. However, these properties can be tuned by copolymerization [5] or combination with other biologically active additives [6,7].

Extensive studies related to bioactive compounds derived from medicinal plants incorporated in PLA by various methods, mostly in the form of extracts or essential oils, have been published. Electrospun PLA mats loaded with *Thymus capitatus* (L.) essential oil (ThymEO) were developed for the treatment of microbial infections. These novel materials showed negligible cytotoxicity, while reductions in viable microbial cells were caused by both the liquid and vapors of the ThymEO released from the mats [8]. Antimicrobial materials were designed using complex emulsion-stabilization approaches. A functionalized PLA substrate was first produced under γ-irradiation (doses of 10 kGy, 20 kGy, and 30 kGy), and bioactive agents such as clove essential oil and argan vegetal oil were incorporated into chitosan that was further immobilized on the surface of the functionalized PLA by a wet treatment involving carbodiimide chemistry [9]. Active agents such as vitamin E and cold-pressed rosehip seed oil were encapsulated into chitosan by the emulsion method and bioplasticizers were incorporated into PLA to develop antimicrobial and antioxidant materials by melt processing [10].

Sage (S) (*Salvia officinalis* from the *Lamiaceae* family) is one of the most popular medicinal plants worldwide. It is used in its natural form or as an extract or essential oil in the food industry, the perfume industry, and mostly in pharmaceutical and medical applications [11,12]. Various species exist in addition to *S. officinalis*, e.g., *S. sclarea* (clary sage), *S. lavandulifolia* (Spanish sage), *S. miltiorrhiza* (danshen), and *S. hispanica* (chia). The antibacterial and generally therapeutic properties of sage are due to the abundance of components such as phenols (rosmarinic, ferulic, and caffeic acid), flavonoids (luteolin, apigenin, and naringenin), and terpenoids (α- and β-thujone, camphor, and 1,8-cineole) [13].

Coconut oil (CO) is an edible oil widely used in Asian and Pacific regions that differs from other edible oils due to its high content of medium chain fatty acids. Lauric acid is the main constituent among the 92% saturated acids found in CO, and this has anti-inflammatory and antimicrobial properties [14]. Virgin coconut oil (VCO) has been shown to act as an antimicrobial and immunomodulatory agent [15].

Nanostructured materials have demonstrated great potential in treating microbial infections [16]. Organoclays are used in the development of biomaterials in various combinations, leading to nanocomposites with desirable properties such as controllable mechanical, swelling, drug release, cytocompatibility or biodegradation behaviors [17,18,19].

The objective of this paper was to develop innovative bioactive materials for potential use in biomedical applications by successful incorporation through melt processing of three commercially available natural additives—sage, coconut oil and nanoclay (I.31PS)—into a PLA biopolymer and to control their structural, morphological, physico-chemical, and biological properties by modulating the type and amount of the components used. Each component was introduced into the neat PLA to induce special features in the non-active and brittle PLA matrix. There were several reasons for incorporating CO, namely to act as a plasticizer for reducing the brittleness of the matrix and enhance its flexibility and also to add antioxidant and antibacterial characteristics to the PLA. The S and nanoclay were intended to provide antioxidant and antibacterial properties, as well as cytocompatibility to the matrix. The novelty of the current study lies in the developed biocomposites, i.e., the synergistic combination of efficient antimicrobial natural components (S, CO, and/or nanoclay) with a PLA biopolymer synthetized from natural resources. The medicinal plant was introduced in a very thin powder form to the molten PLA for a short processing time, avoiding thermal damage. The PLA-based biocomposites were characterized through detailed structural, morphological, physico-chemical, and biological analyses.

## 2. Results and Discussion

### 2.1. ATR-FTIR Spectra Results

The FTIR spectra were analyzed to examine the existence and type of interfacial interaction in the PLA-based composites.

**Figure 1:**
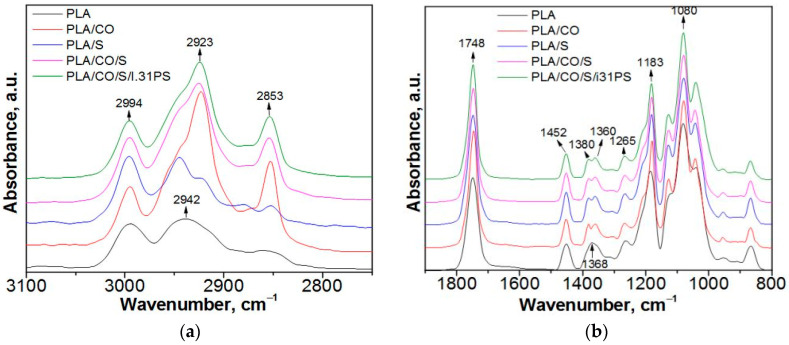
Normalized ATR-FTIR spectra for the PLA-based biocomposites in (**a**) the 3100–2700 cm^−1^ region and (**b**) the 1900–800 cm^−1^ region.

The infrared spectra of PLA biomaterials presented two regions of interest, namely the 3100–2700 cm^−1^ region, assigned to the CH stretching vibrations, and the 1900–800 cm^−1^ region (also called the “fingerprint” region), assigned to the stretching or deformation vibrations of different groups (Figure 1a,b). The FTIR spectra of the neat PLA displayed two bands at 2994 cm^−1^ and 2942 cm^−1^ (CH_3_ and CH_2_, asymmetric stretching vibration), a strong absorbance band at 1748 cm^−1^ (stretching vibrations of amorphous C = O groups), two bands at 1452 cm^−1^ and 1368 cm^−1^ (asymmetric and symmetric bending vibration of C–H from CH_3_), one band at 1265 cm^−1^ (C–O–C stretching vibrations), and two bands at 1183 cm^−1^ and 1080 cm^−1^ (asymmetrical and symmetric valence vibrations of the C–O–C of the aliphatic chain) [20,21].

An increase of the 2994 cm^−1^ signal was observed in the PLA-based biocomposites (Figure 1a), and new signals were detected at about 2923 cm^−1^ and 2853 cm^−1^ (C–H bending vibrations of −CH_3_ and −CH_2_, respectively), corresponding to fatty acids from the coconut oil [22]. In the “fingerprint” region (Figure 1b), there are also modifications: an increase in band intensity from 1748 cm^−1^, 1452 cm^−1^, and 1183 cm^−1^, due to the overlap of specific groups of PLA and coconut oil, and the split of the signal from 1368 cm^−1^, assigned to the symmetric bending vibration of C–H. Additionally, a new signal appears at approximately 1130 cm^−1^, which is attributed to the C–O–C bond stretching vibration of the fatty acids from the coconut oil [23]. No significant differences were observed in the infrared spectra when using the I.31PS nanomer due to the low amount used and the overlap of its specific signals (at about 2920 cm^−1^, 2850 cm^−1^, and 1000 cm^−1^) [24] with the already present stronger bands of the PLA and coconut oil.

### 2.2. Surface Hydrophobicity Evaluation

The water contact angle (WCA) was used to determine the interfacial interactions between water and the PLA-based composites, an important aspect of the physical and chemical processes, such as adhesion and adsorption, used in different applications. A surface with a high water contact angle, i.e., above 90°, is considered hydrophobic, whereas if the contact angle against water is smaller than 90°, the surface is considered hydrophilic [25].

**Figure 2:**
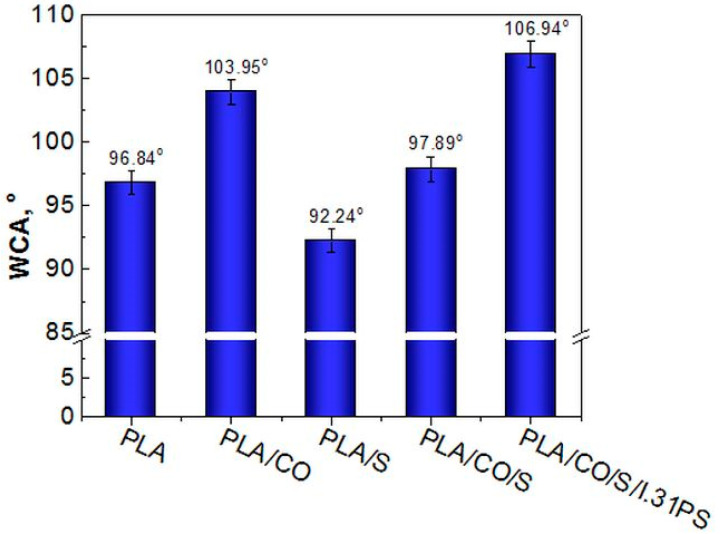
Water contact angle for the neat PLA and the PLA-based biocomposites.

The average contact angle value for neat PLA was about 97° (Figure 2), which is in good agreement with previously reported values in the literature [26,27].

As expected, the hydrophilic character of the PLA/S materials increased compared with that of the neat PLA due to the hydrophilic groups of flavonoids and phenolic compounds, major components in the structure of sage [28,29]. The WCA decreased from 96.84° in neat PLA to 92.24° in the PLA/S (Figure 2).

Blending PLA with CO led to an increase in the WCA from 96.84° up to 103.95° for PLA/CO and to 106.94° for PLA/CO/S/i31PS. Thus, by adding coconut oil as plasticizer, a significant enhancement of the hydrophobic character of the PLA was observed, this effect likely being due to the long hydrocarbon chains of the coconut oil (Figure 2) [30].

The hydrophobicity of particles that contain long hydrophobic chains, such as I.31PS (Figure 2), results from individual chemical functionalization with an organic modifier that adsorbs water and is normally found in pristine clays, minimizing the overall amount of free water to be released [24,31]. This explains why the PLA containing the I.31PS nanomer exhibited an increased hydrophobic character. Hydrophobicity of antimicrobial polymeric materials is a property of interest because investigations have shown the significant impact of hydrophobic interactions between lipid membranes and proteins on biomaterial application efficacy within the body [32,33]. In a study involving moderated hydrophobic polycaprolactone (PCL)/PLA material, round-shaped cells or cluster formations with a monolayer of cells partially adhered to the polymeric surface were observed using SEM, while improved cell viability with the addition of hydroxyapatite and halloysite nanotubes was reported. The protein absorption on the surface of the biomaterial was influenced by variations in wettability, albumin easily adhering to the polyester surface due to its hydrophobic affinity [34].

### 2.3. Morphology Examination

**Figure 3:**
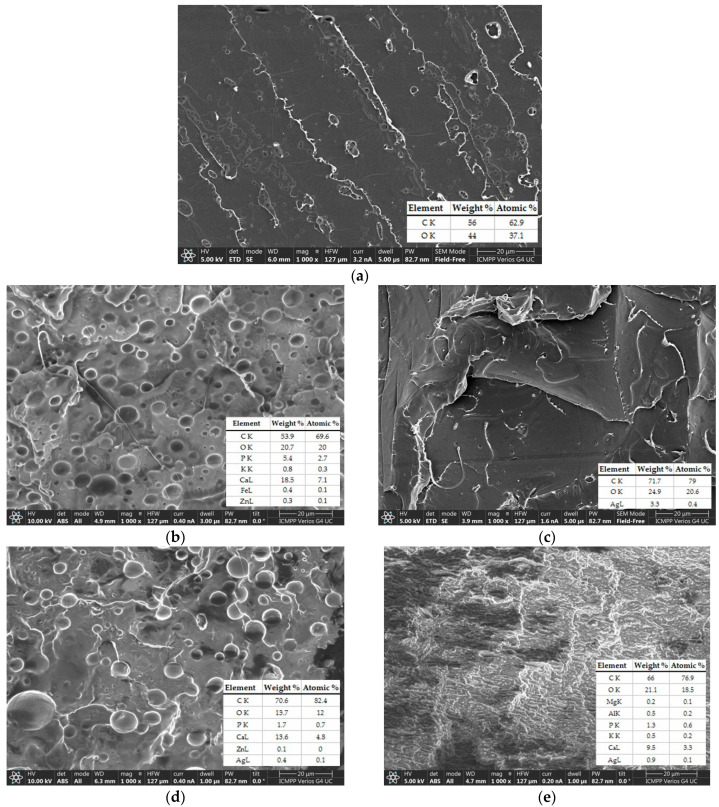
Morphology with elemental composition for the PLA-based biocomposites. Neat PLA (**a**), PLA/CO (**b**), PLA/S (**c**), PLA/CO/S (**d**), and PLA/CO/S/I.31PS (**e**).

**Figure 4:**
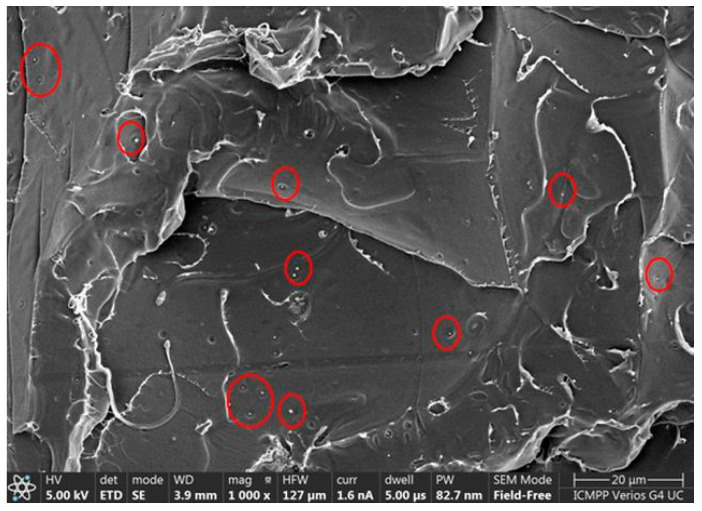
SEM image of the PLA/S material with visualization of sage particle dimensions of ~0.2 μm (red circles).

The morphology of the prepared plasticized PLA and biocomposites fractured in liquid nitrogen was evaluated using SEM (Figure 3). The image displayed in Figure 3a revealed typical aspect of a brittle polymer fracture in the neat PLA, without evidence of heterogeneities. The recorded micrographs showed a uniform phase with well-distributed CO acting as a bioplasticizer (Figure 3b) and sage particles in the PLA matrix (Figure 3c and Figure 4). The images in Figure 3b,d indicated that the CO induced mainly spherical morphologies without any tendency to form phase separations, while the brittle aspect of the neat PLA was no longer observed. A compact morphology can be observed for the PLA/CO/S/I.31PS sample (Figure 3e). In this later case, a very fine homogeneous dispersion of CO into the PLA matrix was observed, indicating good compatibility. This might be explained by the I.31PS nanoclay, which may have improved the interfacial adhesion between the PLA, S, and CO.

Homogeneous distribution without the agglomeration of very fine sage particles of ~0.2 μm within the PLA matrix can be observed in Figure 4.

**Figure 5:**
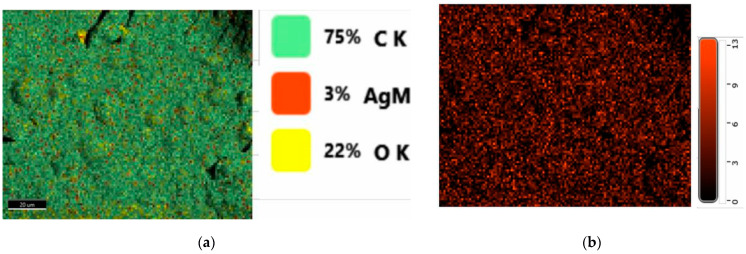
Element overlay (**a**) and visualization of Ag distribution (**b**) within the EDX map of the transverse fractured section of the PLA/S biocomposite. ×1000 magnification (scale bar = 20 μm).

The generation of an EDX map from the fractured surfaces enables a qualitative picture of the element distribution in the biocomposites to be obtained [35]. The quantitative elemental distribution recorded by the EDX evaluation of the developed PLA-based biocomposites is presented in Figure 5a. The uniform localization of Ag within the PLA/S biocomposite is illustrated in Figure 5b, this material presenting the highest amount of Ag in its structure due to the incorporation of 3 wt% sage. The Ag element was also detected in the PLA/CO/S (Figure 3c) and PLA/CO/S/I.31PS (Figure 3d) biocomposites, though in lower amounts. The elemental composition of the most complex material contains Al due to the incorporation of nanoclay.

### 2.4. Mechanical Properties Evaluation

**Figure 6:**
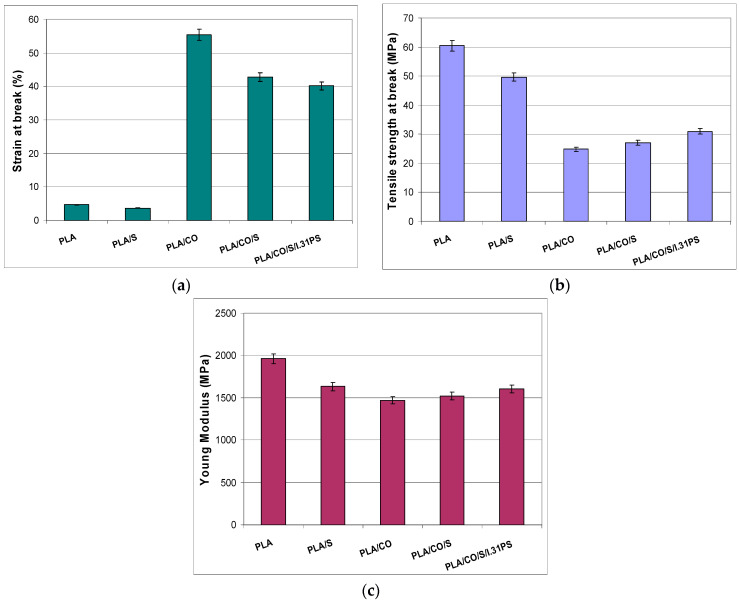
Mechanical properties of the neat PLA and the developed biocomposites: elongation at break (**a**), tensile strength at break (**b**), Young modulus (**c**).

Flexibility is another important feature for biocomposites used in biomedical applications as flexible materials can be easily molded into various shapes. The results corresponding to the tensile tests for the neat PLA and the evaluated biocomposites are displayed in Figure 6. The mechanical properties of neat PLA correspond to a brittle polymer [36], with a low value of elongation at break (EB), high Young modulus (YM), and a tensile strength at break (TS) of 61 MPa, a value close to the 60 MPa reported by the manufacturer [37]. The addition of only 3 wt% S did not affect the elasticity, but decreased the TS and YM by replacing part of the brittle PLA with plant powder. The greatest change in the mechanical properties was observed following the incorporation of 15 wt% CO, which led to an 11-fold enhancement of the EB for the PLA/CO materials compared with the PLA matrix, correlated with an expected reduction in the TS and YM. Similar behavior has recently been demonstrated for PLA/natural and modified castor oil materials [21]. The plasticizing role of the oil was also related to the high flexibility of the other biocomposites in which it was contained, i.e., PLA/CO/S and PLA/CO/S/I.31PS, but in these latter cases an improvement of the stiffness was also observed with a slight increase in YM due to the incorporation of S and nanoclay. The synergistic effect of the components used led to the best mechanical performance of the designed multicomponent biocomposites, a more efficient load transfer under stress conditions being correlated with specific morphologies as displayed in Figure 3, especially for PLA/CO/S/I.31PS.

### 2.5. Antioxidant Activity

**Figure 7:**
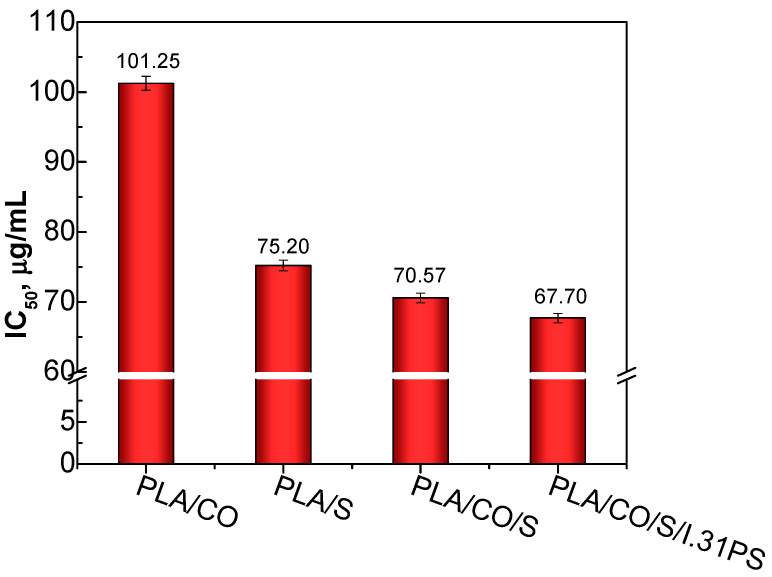
DPPH half maximal inhibitory concentration (IC50) values of the PLA-based biocomposites.

Reactions with the DPPH radical were performed to assess whether the components used in the PLA-based biocomposites would impart their antioxidant activity to the PLA matrix in which they were incorporated. The neat PLA used in this study did not display scavenging activity towards the DPPH radical. Similar results reveal no antioxidant activity in neat PLA [38,39,40]. The antioxidant property was induced by the natural additives incorporated in the PLA matrix. Among the developed biocomposites, the ones containing two or all incorporated natural additives in the PLA matrix displayed the highest antioxidant activity, the strongest being observed in the PLA/CO/S/I.31PS material, as indicated by the very low IC50 concentration (Figure 7). This is probably due to the synergism between the individual components, such as S, CO, and I.31PS.

**Figure 8:**
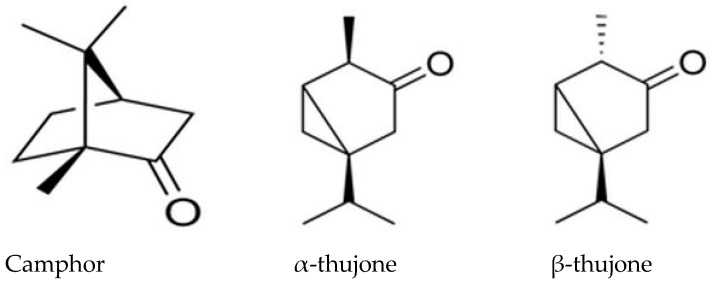
Structures of the main components in sage oil.

It was found that the polyphenols and flavonoid compounds are mainly responsible for the antioxidant and free radical scavenging effects of the sage plant. Phenolic compounds exhibit high antioxidative activity and are usually extracted from sage with ethanol. The phenolic compounds can either stimulate endogenous antioxidant defense systems or scavenge reactive species. The principal components found in the sage oil were camphor (~30%), α-thujone (~40%), and β-thujone (~10%) (Figure 8) [28,29]. This is the reason why the PLA/S composites exhibit antioxidant activity.

It has been demonstrated that silver nanoparticles (AgNP) synthetized using the aqueous extract of common sage (S. officinalis) leaves could act as a DPPH radical inhibitor (IC50 = 170 μm) [41]. Green AgNP synthesized using the alcoholic extract of *S. officinalis* leaves showed a protective role as an antioxidant in nephrotic damage induced by methotrexate (MTX) in adult rabbits [42].

The antioxidant activity of the PLA/CO sample is due to the phenolic acids, such as ferulic acid, *p*-coumaric acid, protocatechuic acid, and vanillic acid, present in the coconut oil structure [43]. However, these are minor components compared with the fatty acids, which represent over 90 wt% of the coconut oil. The fatty acid content of the coconut oil was as follows: the fatty acid for the chain C8–C14 contained approximately 70%, while the chain C16–C18 contained about 30%. This indicates that coconut oil is rich in saturated fatty acids. Lauric acid up to 48% and myristic acid up to 24% have been observed [44,45]. This explains the relatively low antioxidant activity of the PLA/CO composites compared with the PLA/S composites.

The I.31PS nanomer is a high purity surface compatibilized montmorillonite suitable for use in a wide variety of plastics. In addition to traditional inner gallery cation exchange modification, the I.31PS nanomer uses a silane coupling agent as a nanoclay edge treatment agent to enhance dispersion in polymers (Figure 3d) [31,46]. The high antioxidant activity of I.31PS is probably due to the amino groups present in its structure [47,48].

### 2.6. Antimicrobial Assessment

The antimicrobial activity of the PLA-based biocomposites against the gram-positive *Staphylococcus aureus* and gram-negative *Escherichia coli* is presented in Table 1. All the developed biocomposites showed varying degrees of antimicrobial activity, the highest antimicrobial activity being found in the PLA biocomposite containing all of the natural additives, their synergistic effect suppressing the growth of both gram-positive and gram-negative tested bacteria. The PLA used as a reference showed no inhibiting effect on these bacteria. Slightly higher antimicrobial activity has been observed against *S. aureus* for almost all materials, similar findings being reported for poly(vinyl alcohol) (PVA) films containing sage extract or other medicinal plants. Among all the tested plants, sage proved to have the highest antimicrobial efficiency [49]. The variation in the antibacterial activity of the biocomposites might be due to the different active compounds in the structure of the S or CO incorporated into the PLA, by their diffusivity in the growth media, and by differences between the cell wall structures of gram-positive and gram-negative bacteria. The large numbers of different chemical compounds, such as phenols, terpenes, the esters of weak acids, or fatty acids, present in medicinal plants can affect multiple target sites on bacterial cells, inhibiting the growth of microorganisms [50,51]. The antimicrobial activity of *Salvia officinalis* is mainly due to its phenols and has been previously reported by other authors [7,52,53].

Hierholzer and Kabara were the first to report the antimicrobial effect of CO [54]. Other studies have demonstrated that coconut oil exhibit antimicrobial activity against various gram-positive and gram-negative strains, such as *Escherichia vulneris*, *Enterococcer* spp., *Helicobater pylori*, *Staphylococcus aureus*, *Streptococcus mutans*, and *Candia albicans* [55,56,57]. Monolaurin, a monosaccharide present in CO, was found to cause cell shrinkage and cell disintegration in gram-positive cocci [58]. CO contains beneficial medium chain fatty acids (MCFAs), namely lauric acid, capric acid, caprylic acid, and caprioic acid [59]. Among these, the saturated fatty acid lauric acid (LA) (C12), present in virgin coconut oil (VCO), was reported to induce the inhibition of bacterial growth. VCO at a concentration of 200 μL (equal to 0.102% LA) could significantly increase the ability of the macrophage cells to phagocyte *S. aureus* [15].

### 2.7. In Vitro Cytocompatibility Evaluation

**Figure 9:**
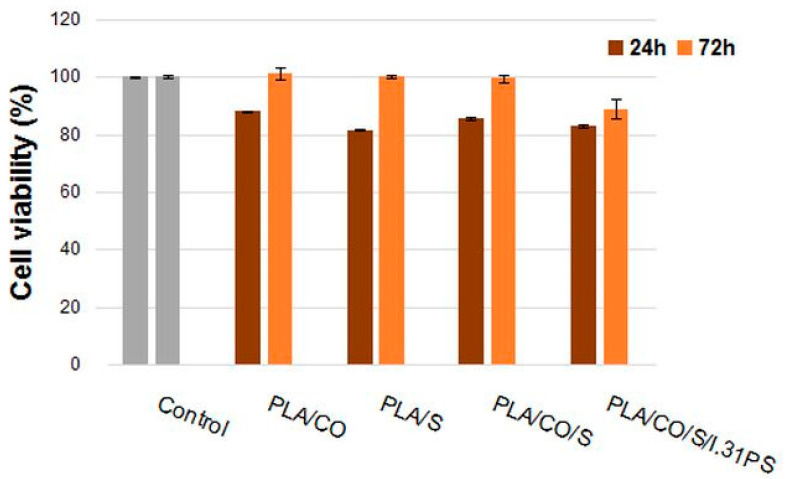
Viability of NCTC mouse fibroblasts cultivated in the presence of PLA/CO, PLA/S, PLA/CO/S, and PLA/CO/S/I.31PS for 24 h and 72 h, evaluated using the MTT assay. Samples reported to control (untreated cells) were considered to have 100% viability. Data are expressed as mean values ± SD (n = 3).

The in vitro cytocompatibility of the biocomposites was assessed using the quantitative MTT assay and a qualitative fluorescent evaluation of the cell morphology. The obtained results highlighted that all of the biocomposites were cytocompatible, the percentages of cell viability being similar to those of the control, except for the PLA/CO/S/I.31PS biocomposite, which presented a slightly lower viability (88.95%) (Figure 9). Each of the samples also induced cell adherence and proliferation on their surface. After 24 h of treatment, the values of cell viability ranged between 81.42% for the PLA/S sample and 88.04% for the PLA/CO sample. After 72 h, an increase in cell viability was observed for all samples, with most values close to that of the control. The highest cell viability value was recorded for PLA/CO (101.11%), followed by PLA/S (100.34%), PLA/CO/S (99.49%), and PLA/CO/S/I.31PS (88.95%).

**Figure 10:**
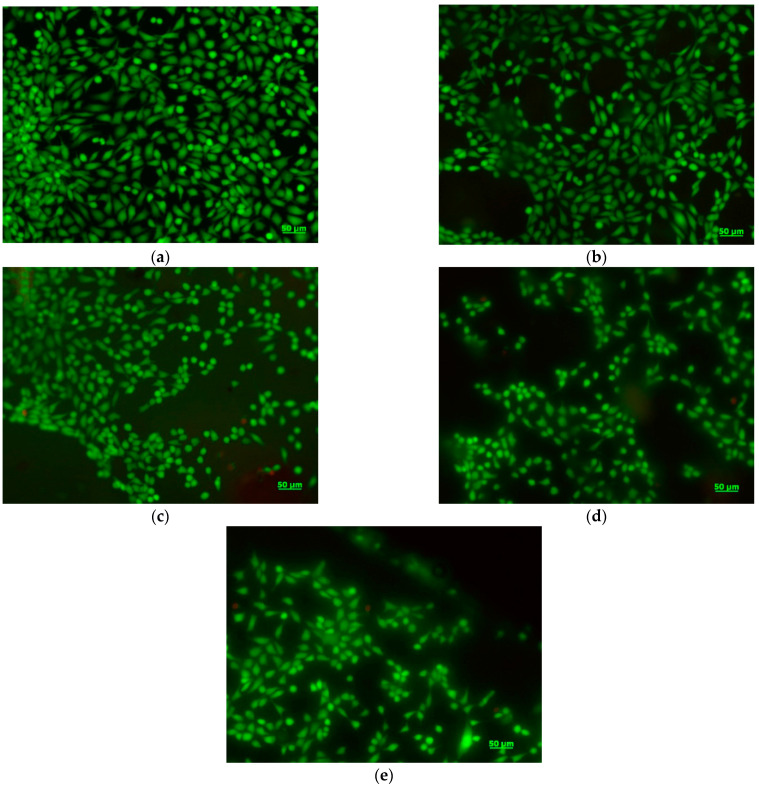
Fluorescent images of NCTC live (green) and dead (red) cells, untreated (**a**) and treated with PLA/CO (**b**), PLA/S (**c**), PLA/CO/S (**d**), and PLA/CO/S/I.31PS (**e**) for 72 h. Scale bar = 50 µm.

Cell morphology and viability were assessed using fluorescence microscopy after staining live cells with calcein (green) and staining dead cells with ethidium homodimer (red). NCTC fibroblasts maintained their viability after 72 h of treatment with all samples, the proportion of dead cells being very low (Figure 10b–e). In addition, treated cells maintained their normal phenotype, similar to that of the control (Figure 10a), with no significant morphological changes. Cell density was also comparable to that of the control, cells being able to adhere and proliferate on the surface of the samples. The morphological observations correlated well with the results obtained by the MTT assay, suggesting the cytocompatibility of all the tested samples.

The preventive effects of VCO against the cytotoxic effect of cyclophosphamide chemotherapeutic medication in the liver and kidney was demonstrated by Nair et al. [60], the positive effect being attributed to linoleic acid and oleic acids, which are essential fatty acids found in CO. The cytological toxicity of VCO was assessed using a mixture of coconut juice, VCO, and methotrexate on MCF-7 breast cancer cells, using a sulforhodamine B (SRB) assay, and its possible anti-cancer effects were evaluated and compared with methotrexate alone, the results showing that concentrations of 10 µg/mL, 30 µg/mL, and 100 µg/mL of VCO and of methotrexate and VCO suppressed cancer cells [61].

Herb extracts or essential oils and nanofillers have been reported to improve the biocompatibility of PLA materials, but no reports dealing with materials incorporating Sage in powder form or coconut oil have been found so far.

Non-cytotoxicity against fibroblastic rabbit cells was recently demonstrated in poly(vinyl alcohol) (PVA) films loaded with extracts obtained from various plants from Romania, such as flowers of the lavender plant (*Lavandula angustifolia*) and leaves of the sage plant (*Salvia officinalis* folium), peppermint plant (*Mentha piperita*), hemp plant (*Cannabis sativa* L.) and verbena plant (*Verbena officinalis*) [49]. Combinations of PLA scaffolds and different herbal extracts for tissue repair and regeneration have been studied by Ilomuanya et al. [62], who developed PLA- (20% w/v) and collagen-based electrospun scaffolds containing silver sulphadiazine (1% w/w; 0.75% w/w) and *Aspalathus linearis* (rooibos) fermented extract (0.025%, 0.1% and 0.5%) for wound healing applications. These scaffolds showed good biocompatibility and provided favorable substrates for the neonatal epidermal keratinocytes to attach to and proliferate. Another study reported the incorporation of nanohydroxyapatite and *Equisetum arvense* extract into PLA composite nanofibrous scaffolds for bone tissue engineering [63]. The scaffolds containing the herbal extract showed excellent cell attachment and promoted the proliferation and osteogenic differentiation of human adipose tissue-derived mesenchymal stem cells by increasing cell viability, ALP activity, and mineralization content. Curcumin, a plant extract with anti-inflammatory, anti-oxidant, and wound-healing properties, incorporated into PLA nanofibrous meshes at a concentration of 0.125 wt% promoted C2C12 mouse myoblast cell attachment and proliferation, and also significantly increased the rate of wound closure in a mouse model compared with PLA nanofibers alone [64]. Our results concerning the biological activity of PLA biocomposites enriched with natural compounds correlate with those of other similar studies, suggesting that our biocomposites possess good cytocompatibility and an ability to promote cell proliferation and, therefore, that they have potential uses in various biomedical applications.

## 3. Materials and Methods

### 3.1. Materials

**Figure 11:**
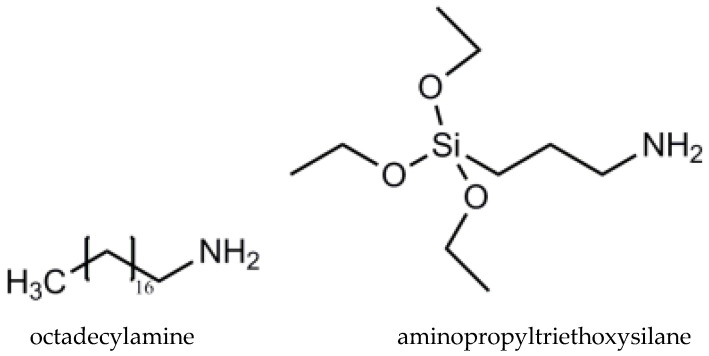
Chemical structure of Nanomer^®^ I.31PS (Adapted with permission from Ref. [31]. Copyright 2023 John Wiley and Sons).

PLA Ingeo^TM^ Biopolymer 2003D was obtained from renewable resources, with a 4% content of D-lactide, supplied by NatureWorks LLC (Minnetonka, MN, USA), in the form of pellets, with a specific gravity of 1.25 g cm^−3^, Mw of 1.43 × 10^5^ g mol^−1^, Mn of 7.54 × 10^4^ g mol^−1^, and a dispersity index (*Đ*_M_) of 1.88 was used as a matrix for the developed biocomposites. PLA has been approved by the FDA for direct contact with biological fluids [4]. Sage (the medicinal plant *Salvia officinalis* L., S) leaves, commercially available from Fares, Orastie, Romania, were ground and sieved to obtain a fine powder of ~0.2 μm. Cold pressed coconut oil (CO) was purchased from SC Solaris Plant SRL, Bucuresti, Romania. Nanomer^®^ I.31PS is a surface-modified montmorillonite nanoclay containing 15–35 wt% octadecylamine and 0.5–5 wt% aminopropyltriethoxysilane (Sigma–Aldrich, Merck, Darmstadt, Germany) (Figure 11).

### 3.2. PLA-Based Biocomposite Processing

PLA is highly hygroscopic and may retain moisture from the atmosphere, leading to degradation of macromolecular chains, reducing the product’s viscosity and overall properties [65,66]. Therefore, before processing, the PLA, sage powder (S), and I.31P nanomer were dried in an oven at 60 °C for 24 h (moisture content < 200 ppm) [21]. The processing of the neat PLA and the polymer blends was performed in a Thermo Scientific™ HAAKE™ PolyLab™ QC mixer (Thermo Fisher Scientific Inc., Vreden, Germany), provided with a mixing chamber of 50 cm^3^, at a temperature of 175 °C and at a counter-rotating screw speed of 60 rotations per minute for 8 min. For the samples containing sage, the PLA was melted and processed for 6 min to a constant torque, and the sage powder was then added and mixed in for 2 more minutes in order to avoid the thermal degradation of the medicinal plant. For the PLA/CO/S and PLA/CO/S/I.31PS blends, the PLA was melt processed together with half the amount of CO (and clay, respectively) for 6 min, while the other half of the CO was manually mixed with sage powder and added to the melted material for a further 2 min. In these latter cases, the CO protected the plant powder from thermal action. The parameters selected for this processing step were based on our previous experiences with the melt processing of various materials based on PLA, the mixing temperature of 175 °C being close to the melting temperature of the PLA matrix, but lower than that required for the initiation of its thermal degradation. A mean speed of 60 rpm was used to reach a homogeneous blend with a uniform distribution of additives [18], while 8 min for total mixing was selected to reach a constant torque and also to avoid the thermal degradation of the resulting material [20,21].

Homogeneous plates of 1 mm and films (thickness ≤ 100 μm) were prepared by hot-pressing the neat PLA and the resulting homogenized mixtures by means of a LabTech LP-20B hydraulic press (LabTech, Samut Prakan, Thailand) at 175 °C with a preheating time of 3 min and a pressing time of 3 min at 140 bars, followed by a sudden cooling of the mold under pressure.

**Figure 12:**
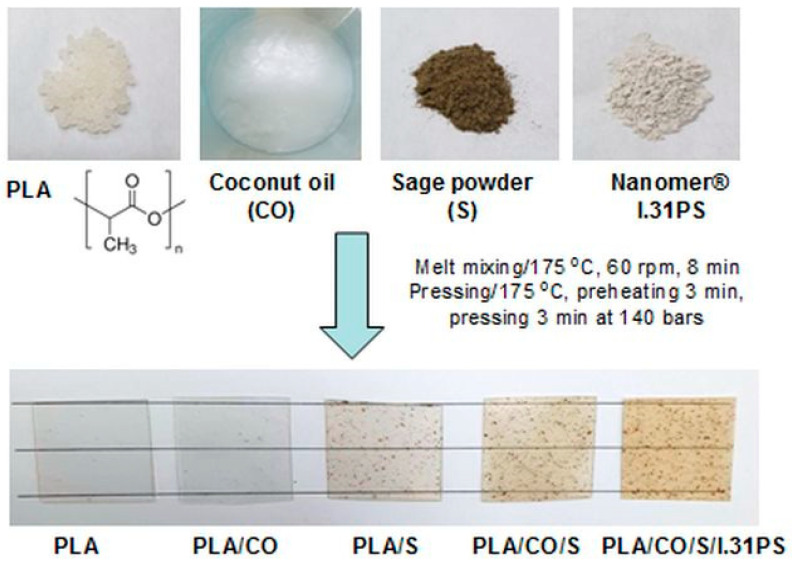
Schematic representation of the developed biocomposites and the visual aspects of the resulting films.

A schematic representation of the developed biocomposites and the visual aspects of the resulting films are presented in Figure 12.

The labeling and compositions of the PLA-based biocomposites containing sage, coconut oil, and the I.31PS nanomer are presented in Table 2. All the materials containing CO were designed with a constant ratio between PLA and CO of 85/15 wt%. In order to avoid exceeding the amount of oil that might migrate from the matrix while also including enough for it to be sufficient for its role as a plasticizer, an amount of 15 wt% for the CO was selected following our reported studies on PLA materials plasticized with epoxidized soybean oil [20] and recently published data regarding the performance of PLA with modified or natural castor oil [21]. An amount of 3 wt% of sage powder was selected based on the results of Gavril et al. [53], who found that increased sage powder content led to issues in melt processing resulting from increased torque due to the agglomeration of the powdered plant, while lower amounts led to PLA/plant materials with no active features. An amount of 3 wt% was chosen for the modified nanoclay I.31PS following our results from the production and characterization of several nanocomposites with a PLA matrix and different types of clays [18].

### 3.3. Investigation Methods

#### 3.3.1. Attenuated Total Reflection-Fourier Transform Infrared Spectroscopy (ATR-FTIR)

The Fourier transform infrared spectra were recorded in ATR mode using a Brucker ALPHA (Platinum ATR) FTIR spectrometer (Bruker Optics GmbH, Ettlingen, Germany) equipped with a diamond crystal in the 3100–800 cm^−1^ region, with a resolution of 4 cm^−1^, using air as background, and with all spectra representing an average of 30 scans. Three recordings were performed for each sample, and an evaluation was made using the average spectrum obtained from these recordings. The processing of the spectra was carried out with the Bruker software OPUS 7.2 (Bruker Optics GmbH, Germany). Prior to each test, a background spectrum was obtained to compensate for the effect of humidity and the presence of carbon dioxide by spectra subtraction.

#### 3.3.2. Water Contact Angle Measurements

Water contact angle (WCA) measurements were used to evaluate whether the PLA-based composite surfaces had hydrophobic or hydrophilic characteristics. The wettability of the surfaces was determined with static contact angle measurements performed using a CAM-200 goniometer (KSV Instruments Ltd., Helsinki, Finland). The water contact angle was determined by the sessile drop method at room temperature and controlled humidity within 5 s after placing 1 µL drops of liquid on the sample’s surface. A video camera recorded the evolution of the droplet shapes, and image analysis software was used to determine the contact angle values. At least 10 measurements were performed on each sample, and the results from three different samples were considered for the statistical determination of the final average value of a material.

#### 3.3.3. Scanning Electron Microscopy (SEM)

The morphologies and the elemental compositions of the developed biocomposites were analyzed by means of a Verios G4 UC scanning electron microscope (Thermo Scientific, Brno, Czech Republic) equipped with an energy dispersive X-ray spectroscopy analyzer (Octane Elect Super SDD detector, EDAX-AMETEK, Mahwah, NJ, USA) [67]. Before analysis, the samples were fractured in liquid nitrogen and dried, then fixed on aluminum stubs with double-adhesive carbon tape and coated with 10 nm gold using a Leica EM ACE200 Sputter coater (Leica Microsystem, Vienna, Austria) to provide material deterioration during electron beam exposure. SEM investigations were performed in high vacuum mode using a secondary electron detector (Everhart-Thornley detector, ETD) at an accelerating voltage of 10 kV.

#### 3.3.4. Examination of Mechanical Properties

The tensile properties, such as tensile strength at break, elongation at break, and Young’s modulus, were examined according to EN ISO 527-2:2011 on a Lloyd LR10K device (Lloyd. Instruments Ltd., Bognor Regis, UK) equipped with a load cell of 500 N. “Dog bone” specimens of 1 mm thickness and 40 mm length cut from the plates obtained by hot-pressing were stretched at a crosshead speed of 10 mm min^−1^ [21]. The average value of at least seven specimens was reported for each composition.

#### 3.3.5. DPPH Radical Scavenging Assay

The radical scavenging activity (RSA) of the PLA-based composites was evaluated using DPPH (2,2-diphenyl-1-picrylhydrazyl), a stable free radical with a violet color that is reduced to a light yellow color under the action of proton donating compounds, a change that can be monitored at 517 nm. Briefly, about 0.5 g composite samples were placed into 20 mL volumes of methanol and shacked at 150 rpm and 25 °C for 24 h. Volumes between 0.5 and 3 mL of the resulting solutions were mixed with 2 mL DPPH solution in methanol (1.5 × 10^−4^ mol/L) and left in the dark for 30 min in closed vials before the UV absorbance was recorded. Equation (1) was used to calculate the radical scavenging activity [40,68]:

%RSA = 100 × (1 − A_sample_/A_control_) (1)

where A_sample_ represents the absorbance of the sample solution and A_control_ represents the absorbance of the DPPH solution with the unmodified sample.

The IC50 value is defined as the concentration at which % RSA reached 50%, and it was calculated using linear regression analysis of the corresponding % RSA values [69].

#### 3.3.6. Antimicrobial Assessment

The antimicrobial activity evaluation (Kirby-Bauer test) was carried out using the agar diffusion method, following the recommendations of the European Committee on Antimicrobial Susceptibility Testing, with some modifications [70]. Two bacteria strains were used in this study: one gram-positive (*Staphylococcus aureus*, PCM2054) and one gram-negative (*Escherichia coli*, ATCC25922). Dimethylsulfoxide (DMSO) was used as a control as well as a solvent for the powdered neat samples of sage and I.31PS, prepared in a 3.0% concentration as in the biocomposites. The tested materials and the paper discs had 6 mm diameters. The filter papers were soaked with 10 µL test samples, then placed in the medium with microorganisms. The tested materials and the samples were placed on Miller-Hinton medium with bacterial and Sabouraud Agar Medium (SAM) and yeast for 5 min after the inoculation of the plates. Miller-Hilton plates were incubated at 35 ± 1 °C and Sabouraud plates were incubated at 26 ± 1 °C in air. The diameters of the inhibition zones (mm) were measured after 18 h. All tests were performed in triplicate. An inhibition zone diameter of more than 3 mm indicated that a tested material was active against the investigated bacteria.

#### 3.3.7. In Vitro Cytocompatibility Evaluation

In vitro experiments were performed on NCTC clone 929 mouse fibroblasts, purchased from the European Collection of Authenticated Cell Cultures (ECACC), using the direct contact method according to the SR EN ISO 10993-5:2009 standard. The cell viability and morphology were evaluated using the 3-(4,5-dimethylthiazol-2-yl)-2,5-diphenyltetrazolium bromide (MTT) assay and the live/dead fluorescent test, respectively [71]. The samples were cut into discs with 5 mm diameters and sterilized under UV light for 4 h. Subsequently, the discs were placed into 24-well culture plates (1 disk/well) and cells were seeded on them at a cell density of 4 × 10^4^ cells/mL using minimum essential medium (MEM) supplemented with 10% fetal bovine serum (FBS) and 1% antibiotics (penicillin, streptomycin, and neomycin). The plates were maintained at 37 °C in a humidified atmosphere with 5% CO_2_ for 24 h and 72 h, respectively, when the two tests were performed. The MTT assay is based on the ability of the mitochondrial succinate dehydrogenases of viable cells to reduce the MTT dye to purple insoluble formazan crystals. After the incubation period, the culture medium was replaced with 0.25 mg/mL MTT solution and the cells were incubated for 3 h at 37 °C. The insoluble formazan crystals were then dissolved with isopropanol by gently stirring for 15 min at room temperature, and the absorbance was measured at 570 nm using a SPECTROstar^®^ Nano microplate reader (BMG, Berlin, Germany). The concentration of converted dye directly correlates to the number of metabolically active cells. The results were calculated using Equation (2) [72]:

% cell viability = OD sample/OD control × 100(2)

The control (untreated cells) was considered to have 100 % viability. All samples were tested in triplicates and data were calculated as mean values ± SD (n = 3).

Cell morphology was examined with fluorescence microscopy using a live/dead assay kit (Molecular Probes, Thermo Fisher Scientific, Waltham, MA, USA). Briefly, after 72 h of incubation in the presence of the samples, the cells were washed with PBS and stained with calcein-AM (2 µM) and ethidium homodimer-1 (4 µM) at room temperature for 30 min. Fluorescent images were acquired using a Zeiss Axio Observer D1 inverted microscope and analyzed with AxioVision 4.6 software (Carl Zeiss Microscopy GmbH., Jena, Germany).

## 4. Conclusions

Three commercially natural additives, namely sage (S), coconut oil (CO), and nanoclay (I.31PS), were successfully incorporated into a PLA biopolymer using the melt processing technique. The morphological, surface, and mechanical properties of PLA biocomposites can be controlled by the addition of S, CO, or a mixture of S, CO, and I.31PS components. Improved flexibility was achieved in all the materials containing CO, which acted as a plasticizer. The highest antioxidant and antimicrobial activities against *S. aureus* and *E. coli* was observed in the PLA/CO/S/I.31PS biocomposite, and this is possibly related to the synergistic effect of the phenolic compounds from the sage structure, the lauric acid from the CO, and the amino group from the montmorillonite nanoclay. The quantitative MTT assay and qualitative fluorescent evaluation of the cell morphology revealed a high degree of cytocompatibility (viability values higher than 80%) in all the tested samples at both exposure times. These results show that the prepared PLA biocomposites with commercial natural additives could be promising bioactive materials for potential biomedical applications.

## Figures and Tables

**Table 1:** Antibacterial activity of the developed biocomposites against *S. aureus* and *E. coli*.

Sample	Mean ± SD Inhibition Zone (mm)	
	*Staphylococcus aureus*	*Escherichia coli*
S	9.8 ± 0.2	8.5 ± 0.1
I.31PS	10.0 ± 0.3	9.2 ± 0.2
PLA/S	9.5 ± 0.3	8.1 ± 0.2
PLA/CO	10.4 ± 0.4	8.9 ± 0.6
PLA/CO/S	10.8 ± 0.3	9.5 ± 0.2
PLA/CO/S/I.31PS	11.6 ± 0.2	10.7 ± 0.3

**Table 2:** Notations and compositions of the developed PLA-based biocomposites.

Sample Code	PLA (wt%)	S (wt%)	CO (wt%)	I.31PS (wt%)
PLA	100	-	-	-
PLA/S	97	3	-	-
PLA/CO	85	-	15	-
PLA/CO/S	82.45	3	14.55	-
PLA/CO/S/I.31PS	79.90	3	14.10	3

## Data Availability

The data presented in this study are available upon request from the corresponding author.
